# Provision of Air Conditioning and Heat-Related Mortality in Texas Prisons

**DOI:** 10.1001/jamanetworkopen.2022.39849

**Published:** 2022-11-02

**Authors:** Julianne Skarha, Amite Dominick, Keith Spangler, David Dosa, Josiah D. Rich, David A. Savitz, Antonella Zanobetti

**Affiliations:** 1 Department of Epidemiology, School of Public Health, Brown University, Providence, Rhode Island; 2 Texas Prison Air Conditioning Advocates, Fulton; 3 Department of Environmental Health, School of Public Health, Boston University, Boston, Massachusetts; 4 Warren Alpert Medical School, Brown University, Providence, Rhode Island; 5 Providence VAMC, Department of Primary Care, Providence, Rhode Island; 6 Center for Health and Justice Transformation, Providence, Rhode Island; 7 Department of Environmental Health, Harvard T.H. Chan School of Public Health, Boston, Massachusetts

## Abstract

**Importance:**

There is a large body of epidemiologic evidence that heat is associated with increased risk of mortality. One of the most effective strategies to mitigate the effects of heat is through air conditioning (AC); Texas regulates the internal temperature of jails to stay between 65 and 85 °F degrees, but these same standards do not apply to state and private prisons.

**Objective:**

To analyze whether heat during warm months is associated with an increased risk of mortality in Texas prisons without AC.

**Design, Setting, and Participants:**

This case-crossover study included individuals who died in Texas prisons between 2001 and 2019. The association of heat in warm months with mortality in Texas prisons with and without AC was estimated. Data analysis was conducted from January to April 2022.

**Exposures:**

Increasing daily heat index above 85 °F and extreme heat days (days above the 90th percentile heat index for the prison location).

**Main Outcomes and Measures:**

Daily mortality in Texas prisons.

**Results:**

There were 2083 and 1381 deaths in prisons without and with AC, respectively, during warm months from 2001 to 2019. Most of the deceased were male (3339 of 3464 [96%]) and the median (IQR) age at death was 54 (45-62) years. A 1-degree increase above 85 °F heat index and an extreme heat day were associated with a 0.7% (95% CI, 0.1%-1.3%) and a 15.1% (95% CI, 1.3%-30.8%) increase in the risk of mortality in prisons without AC, respectively. Approximately 13% of mortality or 271 deaths may be attributable to extreme heat during warm months between 2001 to 2019 in Texas prison facilities without AC. In prisons with AC, a negative percentage change in mortality risk was observed, although the 95% CI crossed zero (percentage change in mortality risk: −0.6%; 95% CI, −1.6% to 0.5%). The estimates in prisons without AC were statistically different than the estimates in prisons with AC (*P* = .05).

**Conclusions and Relevance:**

This study found an average of 14 deaths per year between 2001 to 2019 were associated with heat in Texas prisons without AC vs no deaths associated with heat in prisons with AC. Adopting an AC policy in Texas prisons may be important for protecting the health of one of our most vulnerable populations.

## Key Points

**Question:**

Is heat during warm months associated with an increased risk of mortality in Texas prisons without air conditioning?

**Findings:**

In this case-crossover study of 3464 deaths in Texas prison, a 1-degree increase above 85 °F in prisons without air conditioning was associated with a 0.7% increase in the risk of daily mortality. Approximately, 13% of deaths in Texas prisons during warm months between 2001 and 2019 may be attributable to extreme heat days.

**Meaning:**

These findings suggest that an air conditioning policy for Texas prisons may be an important part of protecting the health of one of our most vulnerable populations.

## Teaser

This case-control study analyzes whether heat during warm months is associated with an increased risk of mortality in Texas prisons without air conditioning.

## Introduction

There is a well-established body of literature that warm temperatures affect health outcomes, including mortality.^1,2^ Extreme heat events, like the 1995 Chicago heat wave, are responsible for hundreds of premature deaths per year in the United States, and an increase in warm ambient temperature is also associated with an increased risk of mortality.^2,3,4,5,6,7^ In the United States, more than 5600 deaths are attributed to heat annually.^8^ Furthermore, climate change is increasing heat severity, frequency, and duration.^9^

Air conditioning (AC) is one of the most successful strategies in mitigating the mortality risk associated with heat exposure. However, current US AC prevalence data available is of low quality and not readily accessible.^10^ Nevertheless, ecological analyses of air conditioning prevalence have consistently found an independent association between AC prevalence and a lower heat-related mortality risk.^11,12,13^ During the 1995 Chicago heat wave, AC was associated with a 3-fold reduced odds of mortality.^4^ Yet AC access varies widely across the United States, especially for some of our most vulnerable populations.^13^

Due to the increased risk for adverse health outcomes caused by the economic and social disenfranchisement of mass incarceration, incarcerated individuals constitute a vulnerable population.^14^ They have a high prevalence of chronic health conditions, and members of minoritized racial and ethnic groups are disproportionately represented.^15,16^ However, there is minimal research on this population’s susceptibility to heat-related illness. Incarcerated persons’ exposure to heat is almost solely regulated through the carceral facility in which they are held. Yet, policies on temperature regulation inside prisons vary widely by state and facility.^17^ For context, jails generally operate as holding facilities before a person receives sentencing, and a stay could be as short as 24 hours; prison facilities are where people serve out a given sentence, and stays generally range from 1 year to tens of decades.^18,19^ In the state of Texas, which has the largest prison population in the nation,^18^ the Texas Commission on Jail Standards requires jails to maintain internal temperatures between 65 and 85 °F (18.3 to 29.4 °C), but this same regulation does not apply to state prisons or private prisons.^20^ Most prison facilities in Texas do not have universal AC. Qualitative reporting from the inside of these prisons during summer months indicates that heat conditions can be extreme.^21^ However, there is limited quantitative research on the health impacts from the lack of AC in Texas prisons. Thus, we aimed to estimate the association between an increase in temperature and risk of mortality in state and private prisons with and without AC in Texas.

## Methods

### Data Sources

The institutional review board at Brown University waived review and the requirement for informed consent since this study does not constitute living human participant research. Mortality data were provided by the Bureau of Justice Statistics Mortality in Correctional Institutions data set for years 2001 to 2019.^22^ This is the only national data set that contains detailed information on mortality of incarcerated adults in state and private prisons, including deaths that occurred outside the facility. Deaths by capital punishment are excluded. Each record includes detailed information about the decedent, such as age, race and ethnicity, and location information for the prison facility in which the person was held. In this study, the only racial and ethnic groups represented were Black, Hispanic, and White. Various correctional professionals complete the record, and they are instructed to report the final cause of death as recorded by a medical examiner or through another official medical investigation. The reproducibility of this data set has been previously described.^23^ Since we were interested in heat, we only focused on deaths that occurred in warm months, which we defined as May, June, July, August, September, and October in each year.

We determined whether someone was likely exposed to AC before their death with facility-specific AC information provided by the Texas Department of Criminal Justice. We then worked with the nonprofit organization Texas Prison Air Conditioning Advocates (TPAA) to categorize prison facilities into either a majority AC group or majority no AC group. We made this choice based on which areas in a facility were air conditioned (such as dormitory areas), information on how recently AC was added to the facility, and lived experience from members of TPAA. We excluded 185 deaths without facility information and 294 deaths that came from 16 facilities missing AC information.

We obtained hourly gridded temperature and humidity data with a 0.125-degree spatial resolution (approximately 12 × 12 km grid) from the North American Land Data Assimilation System.^24,25^ Due to the humid subtropical climate in parts of Texas, we calculated heat index, a measure that incorporates both ambient temperature and relative humidity, using the algorithm provided by the US National Weather Service as implemented in the weathermetrics R package.^26^ We linked mortality data to the gridded heat index databased on the date of death and latitude and longitude of each prison. We used daily maximum heat index (calculated as the maximum hourly value between midnight and 11:00 pm local time) as the main exposure. We also calculated extreme heat days as any day above the 90th percentile heat index for that respective prison location during the 19-year period. For example, if the 90th percentile over a 19-year period for a respective prison location was 95 °F, any day with a temperature above 95 °F would be classified as an extreme heat day.

### Statistical Analysis

We calculated descriptive statistics for mortality and facility characteristics, separated by AC status, and assessed difference by AC status using a χ^2^ test of independence for categorical variables and an independent samples *t* test for age with the interquartile range (IQR) at the 25th and 75th percentiles.

We applied a time-stratified case-crossover analysis to examine the association of daily heat index exposure and extreme heat exposure with mortality in prison facilities with AC and prison facilities without AC. This is a method used extensively in environmental epidemiology to study short-term effects, and it allowed us to compare temperature exposure on the case or death day with temperature exposure on matched control days. Since each death serves as its own control, a key benefit of the case-crossover method is that it controls for all time-invariant confounders, including individual characteristics as well as prison-specific characteristics.^27^ We fitted conditional logistic regression models with a strata variable for each death to estimate the population average change in the risk of daily mortality.^28^

To assess the most influential period of heat exposure prior to death, we applied distributed-lag linear models (DLMs).^29^ DLMs allow us to examine the timing of the exposure response with the advantage of reducing high collinearity of exposures, which is particularly important for modeling the health impacts of temperature. We plotted the lag-response using a natural cubic spline with 4 degrees of freedom at equally spaced values in the log scale over a period of 14 days. We then assessed the nonlinearity of the association between same day (lag 0) heat index and risk of mortality using a natural cubic spline with 3 degrees of freedom. The temperature-mortality association is often V-, U-, or J-shaped, with increases in mortality at temperatures above a heat threshold. Here we defined the baseline temperature (or heat threshold) as 85 °F heat index to replicate the policy on temperature regulation in Texas jails. We assessed model sensitivity by changing the number of degrees of freedom used for the heat index–mortality association model. Finally, to estimate the association of extreme heat with mortality risk, we used DLMs with an indicator variable (0 representing a day without extreme heat day and 1, a day with extreme heat) for the exposure-response association and a natural cubic spline function with 4 degrees of freedom placed at equally spaced values in the log scale for the lag-response relationship. We then used the following equation to test the significance of a difference between the change in risk of mortality in prisons with AC compared with prison without AC^30,31^: 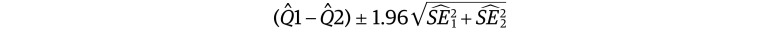
We further calculated the attributable fraction (AF):

*AF_x_* = 1 − exp(−β*_x_*).

The attributable number (AN) of deaths from extreme heat in prisons without AC was calculated with this equation:

*AN_x_* = *n* × *AF_x_*_._

*N* is the total number of deaths in warm months between 2001 and 2019 in Texas prisons.^32^ We present all of our estimates as the mean percentage change in the risk of daily mortality. We performed all statistical analyses in R version 4.1.2 (R Project for Statistical Computing). Statistical significance was set at *P* ≤ .05.

## Results

We found a total of 3464 deaths occurred in Texas prisons during warm months between 2001 most 2019. The majority of the deceased were male (3339 [96%]), and the median (IQR) age at death was 54 years (45-62 years). There were 1381 and 2083 deaths in prisons with AC and without AC, respectively (Table 1). There were similar distributions between the 2 groups by race, sex, and length of time served before death. Compared with the median (IQR) age at deaths in prisons without AC, the median (IQR) age at death in prisons with AC was higher (52 [44-61] years vs 56 [47-63] years; *P* < .001). Comparing facility characteristics (Table 2), there were 10 privately operated prisons with AC and no privately operated prisons without AC; prisons with AC were more likely to be low security; and there was no difference by rurality of prison county or climatic zone.

**Table 1.** Population Characteristics of Decedents in Texas State and Private Prison Facilities From 2001 to 2019 During Warm Months by AC Status of Prison^a^

Characteristic	Mortality in prisons, No. (%)		*P* value^b^
	With AC (n = 1381)	Without AC (n = 2083)	
Sex			
Female	54 (3.9)	71 (3.4)	.50
Male	1327 (96.1)	2012 (96.6)	
Age, median (IQR)	56 (47-63)	52 (44-61)	<.001
Race			
Black	455 (33.0)	652 (31.3)	.43
Hispanic	349 (25.3)	516 (24.8)	
White	577 (41.7)	915 (43.9)	
Time served, y			
<1	249 (18.0)	364 (17.5)	.58
1-10	713 (51.6)	1052 (50.5)	
>10	419 (30.3)	667 (32.0)	

Abbreviation: AC, air conditioning.

^a^ May, June, July, August, September, and October were used as warm months.

^b^ *P* values were derived using a χ^2^ test of independence for categorical variables and an independent samples *t* test for age with the IQR (25th and 75th percentiles).

**Table 2.** Facility Characteristics of Texas State and Private Prison Facilities by AC Status

Characteristic	Prisons, No. (%)		*P* value^a^
	With AC (n = 30)	Without AC (n = 66)	
Facility operator			
State	20 (66.7)	66 (100)	<.001
Private	10 (33.3)	0	
Security level			
Low	25 (83.3)	13 (19.7)	<.001
Medium	4 (13.3)	29 (43.9)	
High	1 (3.3)	24 (36.4)	
Proportion of county is rural^b^			
<10%	5 (16.7)	10 (15.2)	.95
10%-50%	15 (50.0)	32 (48.5)	
>50%	10 (33.3)	24 (36.4)	
Climate^c^			
Humid subtropical	24 (80.0)	50 (75.8)	.84
Dry (arid and semiarid)	6 (20.0)	16 (24.2)	

Abbreviation: AC, air conditioning.

^a^ *P* values were derived using a χ^2^ test of independence.

^b^ As defined in the 2010 US Census Bureau.

^c^ As defined by the Köppen-Geiger climate classification system.

The DLM of heat index and mortality in prison facilities without AC showed increased risk of mortality with an increase in temperature on the same day as the death (lag 0), with no significant excess risks on subsequent days. In prison facilities with AC there was no change in mortality risk (eFigure 1 in the Supplement). Thus, our analysis focused on lag 0. The Figure shows the association between same-day daily maximum heat index above 85 °F and the percentage change in the risk of daily all-cause mortality by AC status; eFigure 2 in the Supplement shows this association with 95% CIs among prison facilities without AC. We found minimal evidence of nonlinearity (eFigure 3 in the Supplement), and so we report these associations as a linear association in Table 3. We found that a 1-degree (1 °F) increase in heat index above 85 °F was associated with a 0.7% (95% CI, 0.1% to 1.3%) increase in the risk of mortality in prison facilities without AC, while in prison facilities with AC, there was a weak inverse association (percentage change in mortality risk: −0.6%; 95% CI, −1.6% to 0.5%). These 2 estimates were statistically different from each another (*P* = .05).

**Figure.**
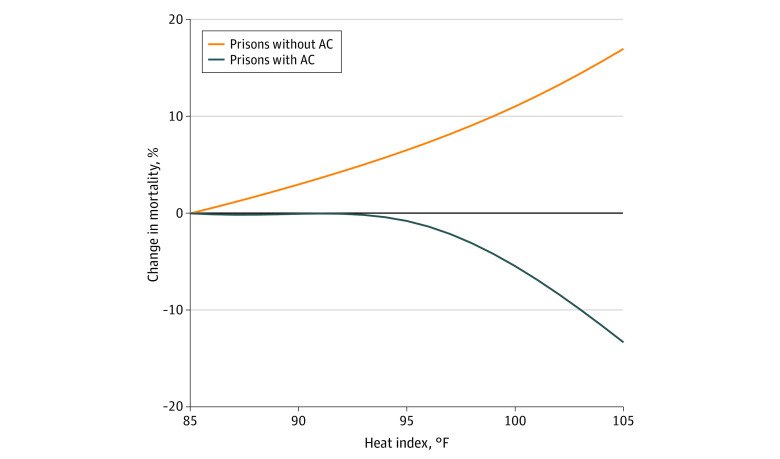
Association Between Same-Day Maximum Heat Index Relative to 85 °F and All-Cause Mortality in Texas Prisons by Air Conditioning (AC) Status From 2001 to 2019, Using a Natural Cubic Spline With 3 Degrees of Freedom

**Table 3.** Percentage Change in Mortality Associated With the Same-Day Maximum Heat Index and Same Day Extreme Heat in Texas Prisons Between 2001 and 2019 During Warm Months^a^

AC status	Continuous heat^b^		Extreme heat^c^	
	Change in mortality (95% CI), %	*P* value^d^	Change in mortality (95% CI), %	*P* value^d^
With AC, reference group	−0.6 (−1.6 to 0.5)	.05	−8.2 (−22.6 to 8.7)	.04
Without AC	0.7 (0.1 to 1.3)		15.1 (1.3 to 30.8)	

Abbreviation: AC, air conditioning.

^a^ May, June, July, August, September, and October were used as warm months.

^b^ Model centered at 85 °F heat index and should be interpreted as the increase in mortality for a 1-degree increase in heat index above 85 °F.

^c^ Extreme heat is defined as a daily heat index above the 90th percentile for the respective prison location and should be interpreted as the increase in mortality for an extreme heat day compared to non-extreme heat day.

^d^ Difference *P* value calculated using the method described in the Statistical Analysis section to determine whether the difference between the 2 groups is statistically significant.

For extreme heat, we similarly found that same day (lag 0) exposure was associated with increased risk of mortality in prison facilities without AC, with no significant excess risks beyond that day, while in prison facilities with AC, there was no change in mortality risk (eFigure 4 in the Supplement). Therefore, we focus on lag 0 also in this analysis. Table 3 shows the association between extreme heat and mean change in risk of all-cause mortality by AC status. We found that an extreme heat day was associated with a 15.1% (95% CI, 1.3% to 31.8%) increase in mortality in prison facilities without AC, while extreme heat was weakly associated with a decrease in risk of mortality in prison facilities with AC (percentage change in mortality risk: −8.2%; 95% CI, −22.6% to 8.7%), a statistically significant difference across facility types (*P* = .04). After calculating the AF, we found approximately 13% of mortality or 271 deaths may be attributable to extreme heat during warm months in Texas prison facilities without AC between 2001 and 2019. This is equivalent to an average of 14 heat-related deaths per year during this time period.

## Discussion

In this case-crossover study we found that both a heat index above 85 °F and extreme heat (day >90th percentile) were associated with increased risk of daily mortality in Texas prison facilities without AC. There was a weak inverse association in Texas prison facilities with AC. These findings suggest that AC may have a protective effect for heat-related mortality. There is life-saving potential if the Texas Department of Criminal Justice applies a similar temperature regulation policy to its prison facilities as it does to its jail facilities.

Previous studies also suggest that AC is protective against heat-related mortality in noninstitutionalized populations. Sera et al^11^ used a longitudinal design from 1972 to 2009 across 211 different cities and found that increased AC prevalence was associated with a 16.7% reduction in heat-related mortality in the United States. Nordio et al^12^ similarly investigated the changing trends in heat-related mortality over time and reported that increased use of AC in the United States was associated with lower relative risk of death. Furthermore, disparities in heat-related mortality have also been linked to AC access.^13^ However, previous research has only focused on noninstitutionalized populations.

To our knowledge, there are no other epidemiologic investigations on the health effects of heat exposure among incarcerated populations. Research on other populations also found in carceral settings shows an elevated risk of mortality from heat exposure. Anderson and Bell^33^ studied extreme heat exposure across 107 US cities over a period of 13 years and found that it was associated with an 8.2% increase in risk of mortality for adults 75 years and older. Older adults are a growing percentage of the incarcerated population due to the mandatory life sentences policies for drug offenses that started in the 1980s, and these individuals may be at particularly increased risk due to their age.^34^

Anderson and Bell^33^ also found that risk of cardiovascular-related mortality among the general population increased by 8.8% during extreme heat events. Adults held in prison are 3.4 times more likely to report having heart-related health problems compared with a standardized age-adjusted noninstitutionalized US population.^15^ In a meta-analysis of extreme temperature exposure and diabetes mellitus, Song et al^35^ reported that heat exposure increased the risk of diabetes-related mortality by almost 14%. Incarcerated individuals are 1.5 times more likely to report having diabetes than the general US population.^15^ Finally, certain medications, particularly psychotropic drugs, affect thermoregulation and can increase the risk of heat-related illness.^36^ This is an important risk factor for incarcerated populations, given that 66% of adults held in state and federal prisons reported taking prescription medication in the 2011 to 2012 National Inmate Survey.^15^

We found that 13% of mortality during warm months may be attributable to extreme heat in prisons without AC in Texas. This is approximately a 30-fold increase in heat-attributed deaths when compared with estimates (0.35%-0.44%) in the general US population.^2,8^ This difference might be explained by common building materials for prisons, such as concrete and steel, which may exacerbate heat conditions. These facilities may also be overpopulated, which could make spaces hotter from individual body heat. Finally, use of other cooling resources, such as AC, cold water, fans, and shade, may be more accessible to the general population than to an incarcerated population.^21^

### Limitations

This study has limitations. Due to the limited sample size, we could not look at effect modification by important characteristics, such as age and cause of mortality. This would have been more informative about who is most at risk of mortality during heat events. This also limited our ability to look at more extreme events, such as days with heat greater than the 95th percentile, or consecutive extreme heat days, which may be a better measure for extreme heat exposure. Another key limitation is that there may be some important differences between prison facilities with AC and those without AC besides AC itself. For example, facilities that are designed for medical treatment or drug treatment centers are more likely to have AC than a standard prison facility. These treatment centers may provide better access to medical care, which could explain the lack of association between heat and mortality rather than AC. Nevertheless, even if we did not fully capture the association of heat and risk of mortality in prison facilities with AC, the association between heat and increased risk of mortality in prison facilities without AC can be viewed on its own without comparison to facilities with AC. Another important limitation is that although we did not find an association between increasing temperatures and increased risk of mortality among prisons with AC, this may be partly due to the 85 °F threshold we used (to compare with the jail policy). Previous research indicates that even moderate amounts of heat have health impacts at the population scale and thus the total heat mortality burden may not be adequately captured using a threshold of 85 °F.^2^ Further research could focus on determining the minimum mortality temperature within these settings.

Determining the AC status of a prison can also be difficult. There may be certain parts of a facility that have AC, such as a medical unit, staff area, or geriatric dorm, and we did not have information on whether the person who died was held in an area of a prison facility with AC. Thus, there may be deaths in the prisons without AC group that were actually exposed to AC. However, we expect that removing those deaths would only increase our effect estimates. Finally, similar to most epidemiologic studies on ambient heat, our analysis was limited by a lack of data on individual-level temperature exposures experienced. We expect that our use of outdoor heat index reliably captures the day-to-day variability in thermal conditions experienced inside prison environments broadly, but individual-level exposures undoubtedly vary both between prisons (from factors such as building materials, ventilation, and non-AC heat mitigations used at each facility, if any) and within prisons (from individual-scale variability in activities and physical locations within facilities).

## Conclusions

In this case-crossover study, we found that 13% of mortality (or 271 deaths) may be attributed to extreme heat during warm months in Texas prisons without universal air conditioning between 2001 and 2019. Our findings have important health implications for the approximately 160 000 individuals held in Texas prison facilities annually as well as the thousands of correction officers, nurses, and other staff that work in these settings.^18^ Adopting a universal AC policy in all Texas prisons may be necessary for the health of one of the most vulnerable populations in the United States.

## Notes

Supplement. **eFigure 1.** Mortality Lag-Response Curve for a 1-°F Increase Above 85°F Heat Index During Warm Months in Texas by AC Status **eFigure 2.** Association Between Same-Day Maximum Heat Index Relative to 85°F and All-Cause Mortality in Texas Prisons Without From 2001 to 2019, Using a Natural Cubic Spline With 3 Degrees of Freedom **eFigure 3.** Sensitivity Analyses for Modeling the Association Between Heat Index and Mortality in Texas Prisons Using Different Degrees of Freedom for the Natural Cubic Spline **eFigure 4.** Mortality Lag-Response Curve for an Extreme Heat Day in Texas by AC Status
