# Error in Methods and Article Information

**DOI:** 10.1001/jamanetworkopen.2022.49232

**Published:** 2022-12-08

In the Original Investigation “Provision of Air Conditioning and Heat-Related Mortality in Texas Prisons,”^1^ published November 2, 2022, there was an error in the Methods and Article Information sections. The group Texas Prison Air Conditioning Advocates (TPAA) should have appeared as Texas Prisons Community Advocates (TPCA). This article has been corrected.^1^
