# Supplementary material for: Provision of Air Conditioning and Heat-Related Mortality in Texas Prisons
Source: JAMA Netw Open. 2022 Nov 2;5(11):e2239849. doi: 10.1001/jamanetworkopen.2022.39849 (PMC9631100; doi:10.1001/jamanetworkopen.2022.39849)
Supplement: Supplement. — eFigure 1. Mortality Lag-Response Curve for a 1-°F Increase Above 85°F Heat Index During Warm Months in Texas by AC Status eFigure 2. Association Between Same-Day Maximum Heat Index Relative to 85°F and All-Cause Mortality in Texas Prisons Without From 2001 to 2019, Using a Natural Cubic Spline With 3 Degrees of Freedom eFigure 3. Sensitivity Analyses for Modeling the Association Between Heat Index and Mortality in Texas Prisons Using Different Degrees of Freedom for the Natural Cubic Spline eFigure 4. Mortality Lag-Response Curve for an Extreme Heat Day in Texas by AC Status [file jamanetwopen-e2239849-s001.pdf]

Supplemental Online Content

Skarha J, Dominick A, Spangler K, et al. Provision of air conditioning and heat-related mortality in Texas prisons. *JAMA Netw Open.* 2022;5(11):e2239849.
doi:10.1001/jamanetworkopen.2022.39849

eFigure 1. Mortality Lag-Response Curve for a 1-°F Increase Above 85°F Heat Index During Warm Months in Texas by AC Status

eFigure 2. Association Between Same-Day Maximum Heat Index Relative to 85°F and All-Cause Mortality in Texas Prisons Without From 2001 to 2019, Using a Natural Cubic Spline With 3 Degrees of Freedom

eFigure 3. Sensitivity Analyses for Modeling the Association Between Heat Index and Mortality in Texas Prisons Using Different Degrees of Freedom for the Natural Cubic Spline

eFigure 4. Mortality Lag-Response Curve for an Extreme Heat Day in Texas by AC Status

This supplemental material has been provided by the authors to give readers additional information about their work.

© 2022 Skarha J et al. *JAMA Network Open.*

eFigure 1. Mortality Lag-Response Curve for a 1-°F Increase Above 85°F Heat Index During Warm Months in Texas by AC Status

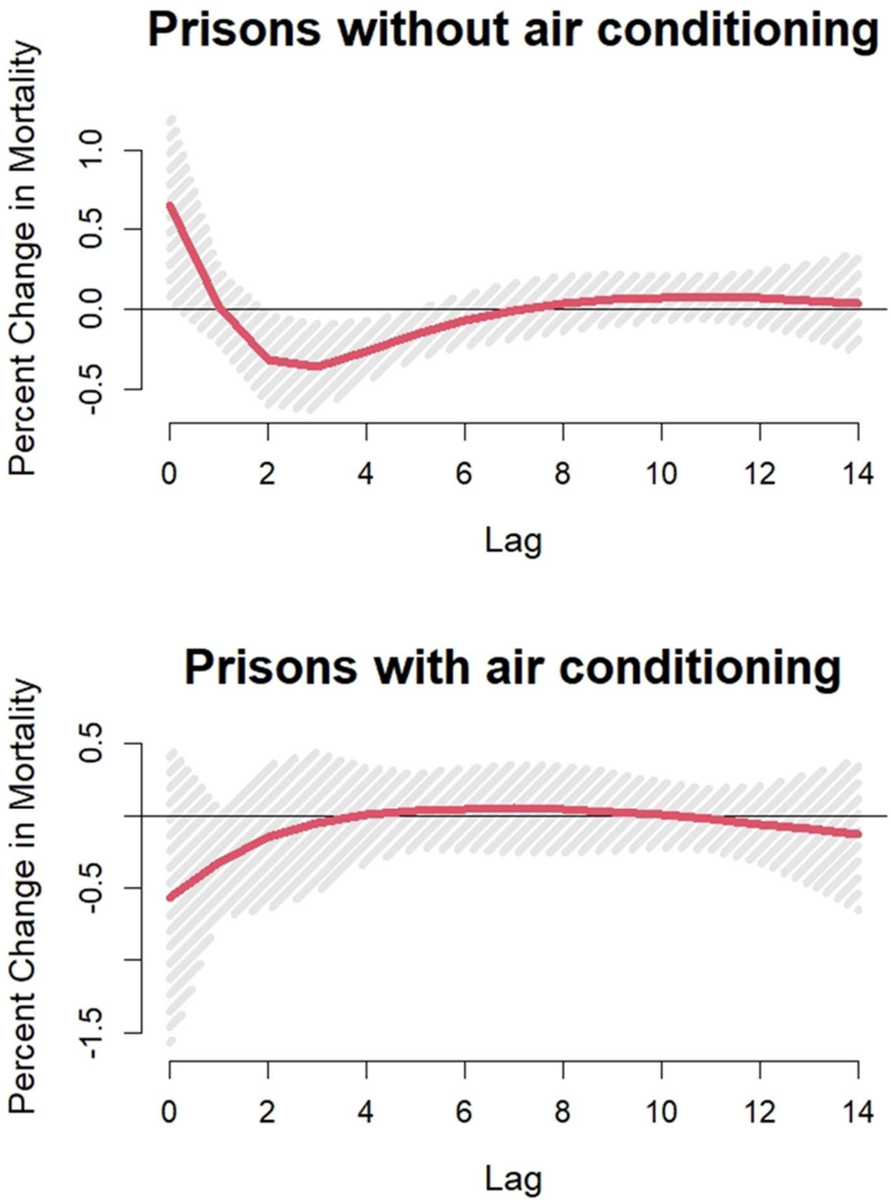

© 2022 Skarha J et al. *JAMA Network Open.*

eFigure 2. Association Between Same-Day Maximum Heat Index Relative to 85°F and All-Cause Mortality in Texas Prisons Without From 2001 to 2019, Using a Natural Cubic Spline With 3 Degrees of Freedom

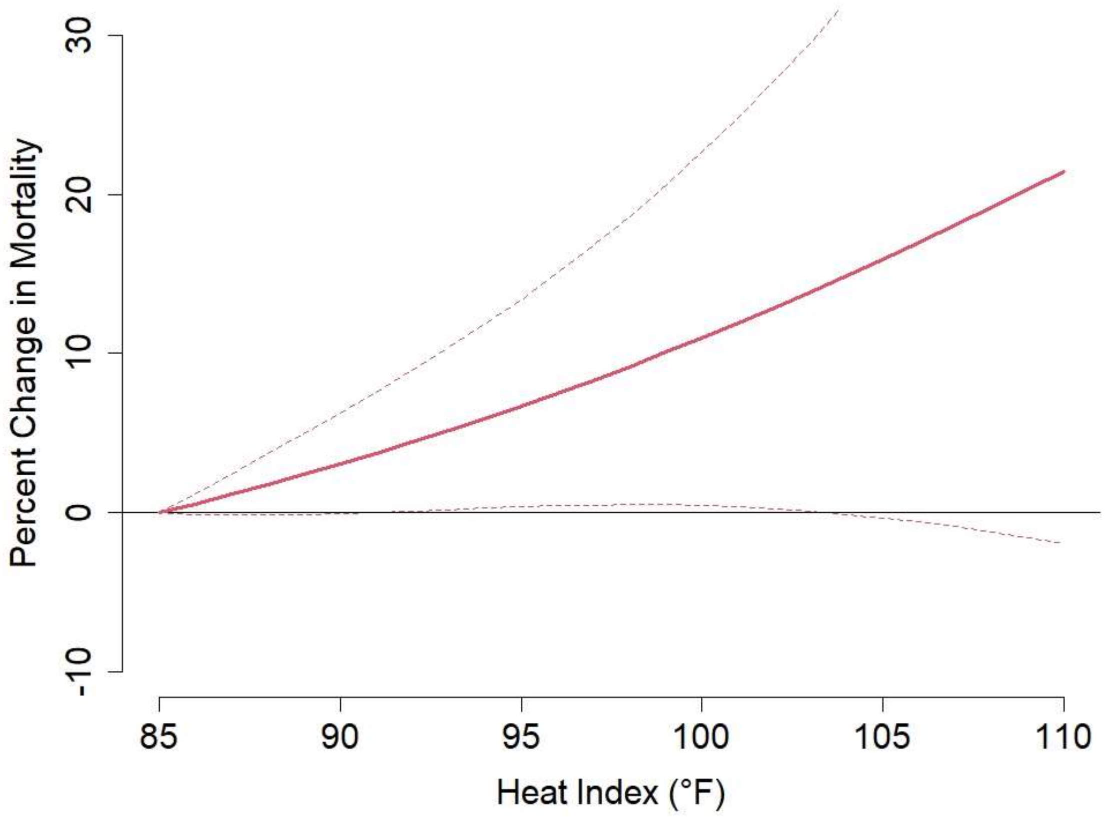

© 2022 Skarha J et al. *JAMA Network Open.*

eFigure 3 . Sensitivity Analyses for Modeling the Association Between Heat Index and Mortality in Texas Prisons Using Different Degrees of Freedom for the Natural Cubic Spline

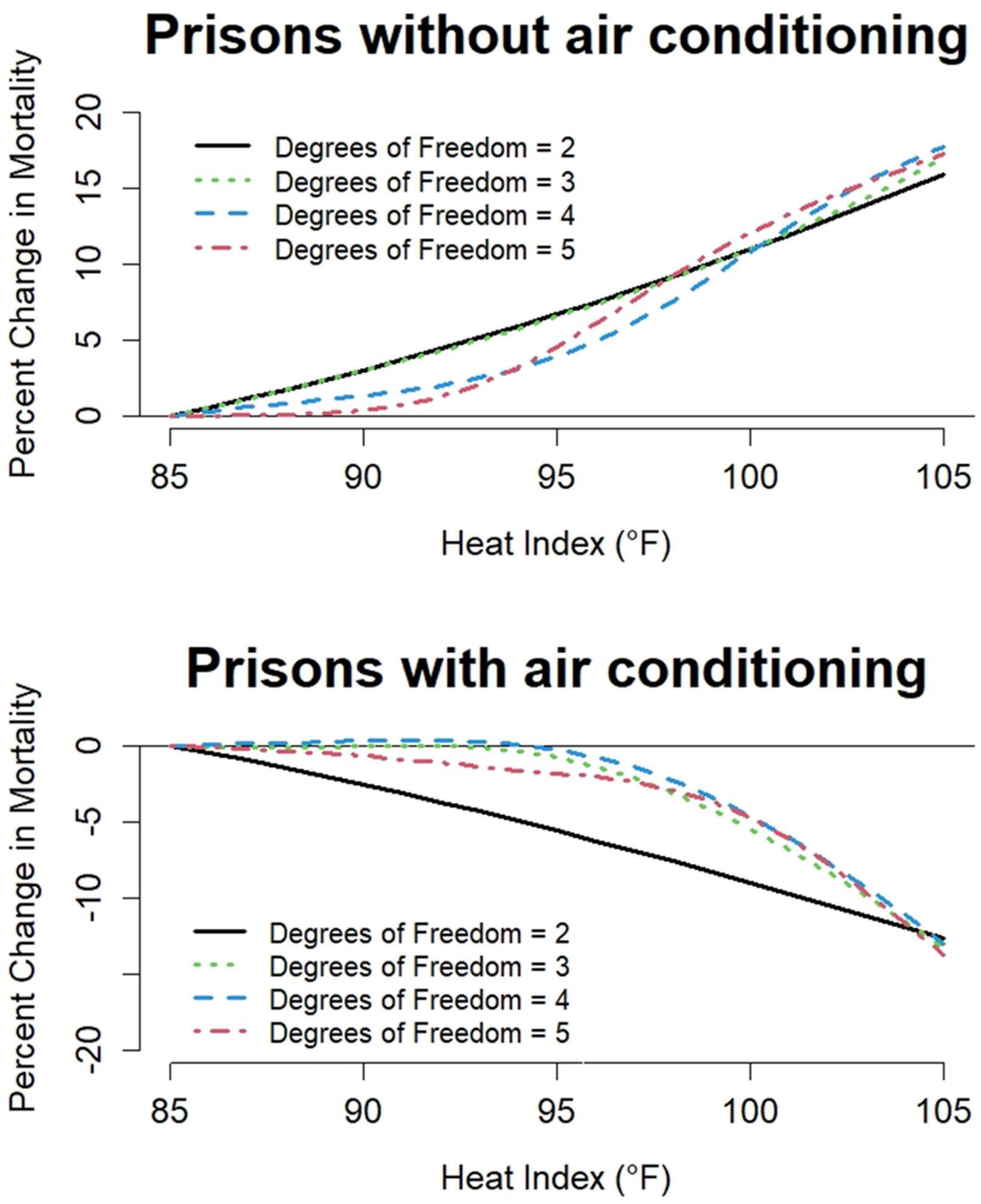

© 2022 Skarha J et al. *JAMA Network Open.*

eFigure 4. Mortality Lag-Response Curve for an Extreme Heat Day in Texas by AC Status

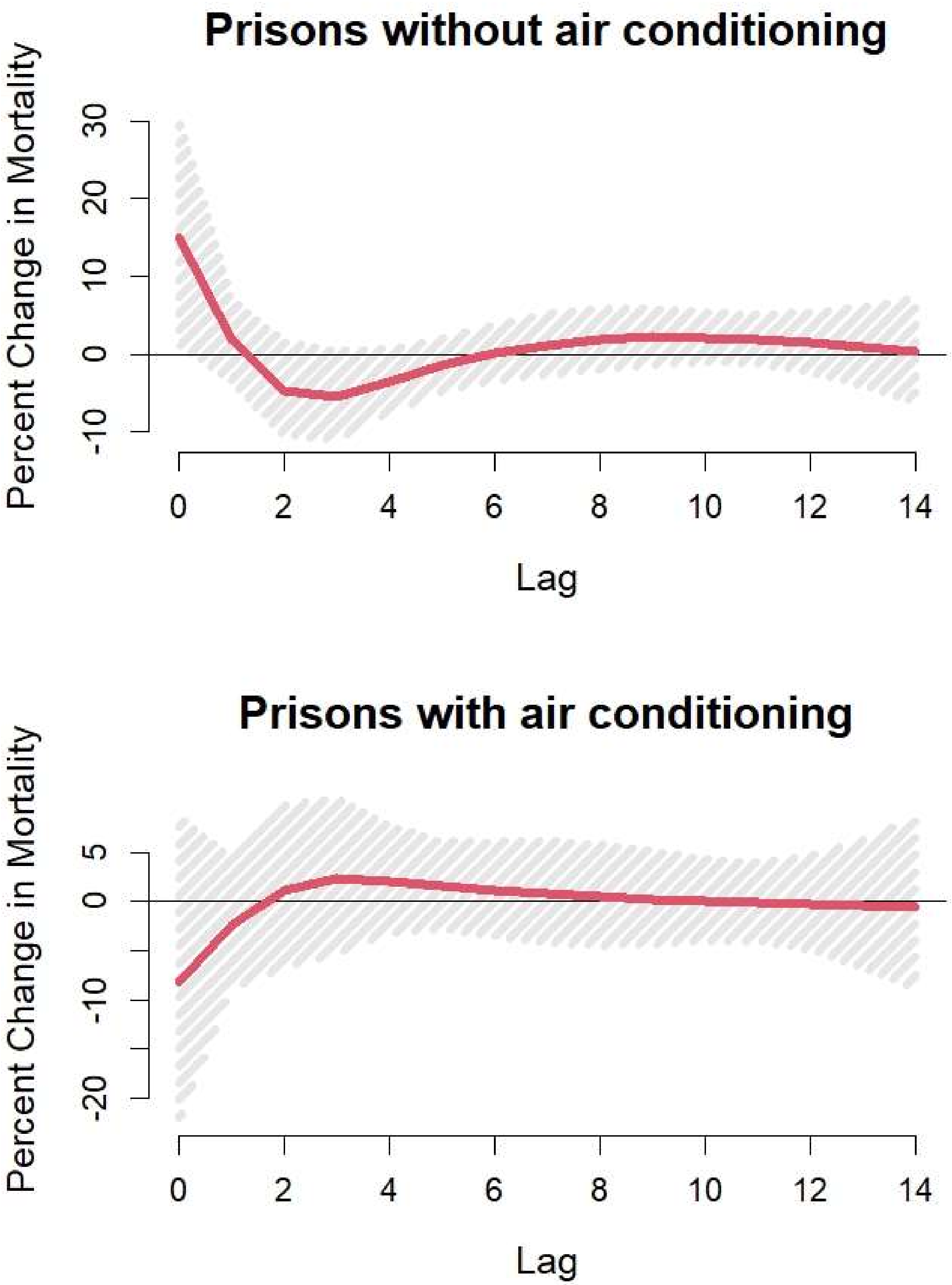

© 2022 Skarha J et al. *JAMA Network Open.*
